# The administration of exogenous HSP47 as a collagen-specific therapeutic approach

**DOI:** 10.1172/jci.insight.181570

**Published:** 2025-02-06

**Authors:** Roberta Besio, Nadia Garibaldi, Alessandra Sala, Francesca Tonelli, Carla Aresi, Elisa Maffioli, Claudio Casali, Camilla Torriani, Marco Biggiogera, Simona Villani, Antonio Rossi, Gabriella Tedeschi, Antonella Forlino

**Affiliations:** 1 Department of Molecular Medicine, Biochemistry Unit, University of Pavia, Pavia, Italy.; 2 Department of Veterinary Medicine and Animal Sciences (DIVAS), University of Milan, Lodi, Italy.; 3 CIMAINA, University of Milan, Milano, Italy.; 4 Department of Biology and Biotechnology, and; 5 Department of Public Health, Experimental and Forensic Medicine, University of Pavia, Italy.

**Keywords:** Cell biology, Therapeutics, Bone disease, Collagens

## Abstract

The proof of principle of the therapeutic potential of heat shock protein 47 (HSP47) for diseases characterized by defects in collagen I synthesis is here demonstrated in osteogenesis imperfecta (OI), a prototype of collagen disorders. Most of the OI mutations delay collagen I chain folding, increasing their exposure to posttranslational modifications that affect collagen secretion and impact extracellular matrix fibril assembly. As a model, we used primary fibroblasts from OI individuals with a defect in the collagen prolyl 3-hydroxylation complex, since they are characterized by the synthesis of homogeneously overmodified collagen molecules. We demonstrated that exogenous recombinant HSP47 (rHSP47) is taken up by the cells and localizes at the ER exit sites and ER-Golgi intermediate compartment. rHSP47 treatment increased collagen secretion, reduced collagen posttranslational modifications and intracellular collagen retention, and ameliorated general ER proteostasis, leading to improved cellular homeostasis and vitality. These positive changes were also mirrored by an increased collagen content in the OI matrix. A mutation-dependent effect was found in fibroblasts from 3 probands with collagen I mutations, for which rHSP47 was effective only in cells with the most N-terminal defect. A beneficial effect on bone mineralization was demonstrated in vivo in the zebrafish *p3h1^–/–^* OI model.

## Abstract 2

Exogenous HSP47 reduces collagen overmodification favouring its secretion and extracellular matrix deposition in osteogenesis imperfecta: a new collagen specific therapeutic approach.

## Graphical abstract

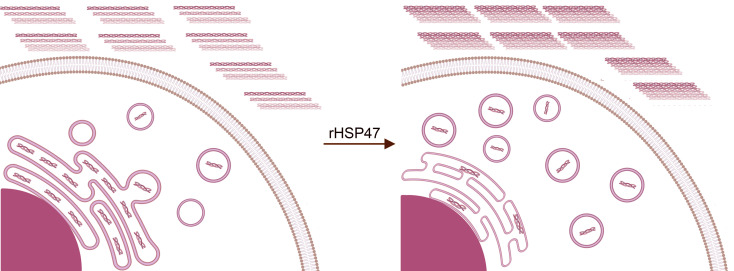

## Introduction

Collagen type I, the most abundant protein in the human body, provides tissues with the necessary structural and functional support. Collagen is synthetized as procollagen molecule in the ER where 2 proα1 chains and 1 proα2 chain assemble from the C-terminal end to form a trimer with a central clockwise triple helical domain flanked by 2 globular N- and C-terminal propeptides (1). In the ER, procollagen I chains undergo posttranslational modifications (PTMs), including hydroxylation of triple helical and telopeptide lysine residues, performed by lysyl hydroxylase-1 and -2, respectively (2). Proline residues are also hydroxylated at C-4 and C-3 by prolyl 4-hydroxylase B and prolyl 3-hydroxylase complexes, respectively, the latter composed of prolyl 3-hydroxylase 1 (P3H1) associated in a 1:1:1 ratio with cartilage-associated protein (CRTAP) and cyclophilin B (CyPB) (2). Some hydroxylysines are then glycosylated by hydroxylysyl-galactosyltransferase and galactosyl-hydroxylysyl-glucosyltransferase that transfer a galactose unit to hydroxylysine residues and a glucose unit to galactosyl-hydroxylysine residues, respectively (3, 4). Then, procollagen leaves the ER and moves to the Golgi intermediate compartment (ERGIC) in coat protein complex 2 (COP2) vesicles (5). Large cargo molecules such as collagen require specialized factors to promote their packaging and exit from the ER. Kelch-like protein 12 (KLHL12) facilitates collagen transport by binding to a COP2 component, SEC31, and triggering its monoubiquitinylation (6). Once in the Golgi, procollagen is transported in post-Golgi tubular saccular carriers by the detachment of large regions of the *trans*-Golgi (7–9) and bundles of procollagen are then released to form secretory vacuoles (10). Following procollagen secretion, the pericellular removal of N- and C-terminal propeptides allows the self-assembling of mature collagen molecules in extracellular matrix (ECM) fibrils (2, 10). Chaperones and several multiprotein complexes are involved in collagen biosynthesis (11), and among them heat shock protein 47 (HSP47) has multiple roles during the process. In the ER, HSP47 interacts with the Gly-X-Arg motif and weakly with the Gly-Pro-Hyp motif at the N-terminal region of nascent procollagen polypeptide chains to help their folding and recognizes the folded triple helical conformation, preventing local triple helix unfolding and intracellular aggregation (12). HSP47 also has a relevant role in secretion since it bridges procollagen to the ER transmembrane protein TANGO1, a key player in the formation of the COP2 vesicles used by procollagen to leave the ER (11).

Altered collagen I intracellular synthesis, secretion, and extracellular processing and assembly characterize both physiological conditions such as aging, and pathological conditions like vitamin C deficiency, homocystinuria, osteoporosis, and genetic diseases like Ehlers-Danlos syndrome and osteogenesis imperfecta (OI) (13–16). OI is usually referred to as brittle bone disease, since the primary clinical manifestations are skeletal deformity and fragility associated with reduced bone mass. Nevertheless, extraskeletal manifestations have been reported that include pulmonary function impairment, cardiac valve abnormalities, muscle weakness, blue sclerae, hearing loss, dentinogenesis imperfecta, and ligamentous laxity (14, 17). OI is a family of heritable collagen type I–related disorders, caused by defects in different genes, and it is often associated with matrix collagen insufficiency caused either by quantitative defect, due to reduced normal collagen I synthesis, or by intracellular retention of collagen molecules with an altered structure.

Currently, there is no effective therapy available for OI. Most of the treatments focus primarily on conservative and surgical intervention to improve the patients’ quality of life and pharmacological therapy is still for the most part based on bisphosphonate administration (18, 19). Despite their positive effects on bone mass, bisphosphonates decrease bone turnover and studies on humans and mice have raised concerns about the effect of high cumulative doses of the drug on bone remodelling and healing**,** especially at pediatric age (20–22). The development of anabolic approaches has been more recently proposed based on the possibility to increase collagen synthesis and thus bone mass. To this aim, an anti-sclerostin antibody that inhibits the negative regulator of the WNT pathway in osteoblasts and was recently approved for osteoporosis treatment by the EMA and FDA was used in OI animal models where it improved bone strength and microarchitecture, and reduced axial and long bone fractures (23–25). The same drug in phase IIa and IIb clinical trials has shown an increase in markers of bone formation, along with a decrease in bone resorption markers and a bone mineral density gain at the lumbar spine. Unfortunately, cardiovascular safety concerns for major cardiovascular events were raised during the clinical trials (26), underscoring the need for new and safer therapeutic molecules.

Collagen biosynthesis and secretion strongly depend on the proper expression and function of the collagen-specific molecular chaperone HSP47 and hence it is tempting to hypothesize its possible use as an OI drug. Unlike other heat shock proteins and other ER-resident proteins involved in collagen folding and maturation, HSP47 does not associate with other known client proteins (27–29). The chaperone is expressed in all collagen-synthesizing cells, and constitutive expression levels correlate strictly with the amounts of collagen being synthesized. Administration of exogenous HSP47 could potentially exert a specific effect on collagen secretion in all collagen-producing cells, thus targeting all OI-affected tissues, unlike other proposed treatments.

Here we evaluated the therapeutic potential of a recombinant form of HSP47 (rHSP47) on OI patients’ fibroblasts. As prototype defects for a first proof of concept of the efficacy of HSP47 treatment in the presence of collagen with abnormal structure, we chose the recessive OI types VII (OMIM #610682) and VIII (OMIM #610915) caused by mutations in *CRTAP* and *P3H1*, respectively (30, 31). These mutations, unlike the classical OI dominant cases associated with mutations in the collagen genes, cause the synthesis of collagen molecules with similar level of PTMs and thus constitute the ideal substrate for testing a drug acting on collagen folding and secretion.

The drug effect on collagen deposition was also evaluated in fibroblasts from patients with collagen I mutations in different positions along the chain. Lastly, bone mineralization was assessed upon rHSP47 treatment in vivo in a zebrafish OI model.

## Results

### rHSP47 reduces intracellular procollagen I retention.

**Figure 1:**
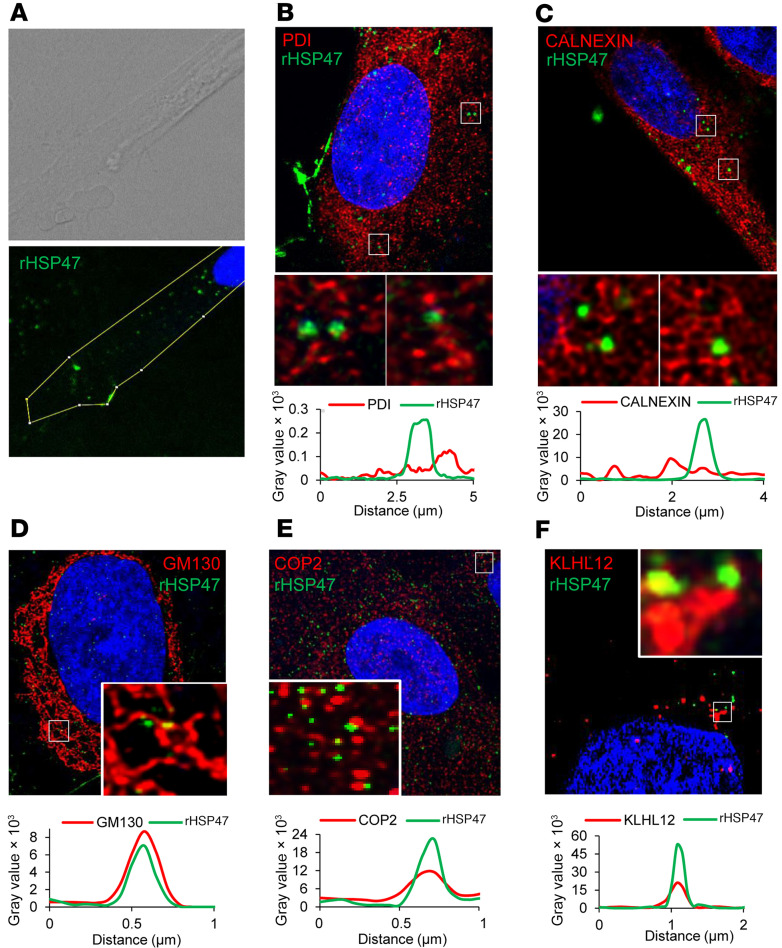
Recombinant heat shock protein 47 (rHSP47) is taken up by human fibroblasts. (**A**) The intracellular uptake and presence of rHSP47-GFP is shown by bright-field and immunofluorescence (*n* = 3, representative image is shown). (**B** and **C**) Representative immunofluorescence images of primary wild-type fibroblasts incubated with rHSP47-GFP and stained for the ER markers protein disulfide isomerase (PDI) and calnexin (*n* = 3), (**D**) with the *cis*-Golgi marker Golgi matrix protein 130 (GM130), and with markers of the secretory vesicles (*n* = 3) (**E**) coat protein complex 2 (COP2) (*n* = 3) and (**F**) kelch-like family member 12 (KLHL12) (*n* = 3). Colocalization of rHSP47-GFP with *cis*-Golgi and secretory vesicles, but not with ER, is indicated by the overlapping peaks in the graphs. Nuclei were counterstained with DAPI. Original magnification, ×63 (insets further magnified ×4.5).

Exogenous rHSP47 cellular uptake was first demonstrated in control human fibroblasts by confocal microscopy using rHSP47 covalently linked to GFP (rHSP47-GFP) (Figure 1A). Surprisingly, coimmunolabeling showed no localization of rHSP47-GFP either with the ER lumen marker protein disulfide isomerase (PDI) or the ER membrane marker calnexin (Figure 1, B and C). Colocalization of rHSP47-GFP with specific sites of the secretory pathway, namely *cis-*Golgi (*cis*-Golgi matrix protein 130, GM130) and secretory vesicles (COP2 and KLHL12), was instead found (Figure 1, D–F).

**Figure 2:**
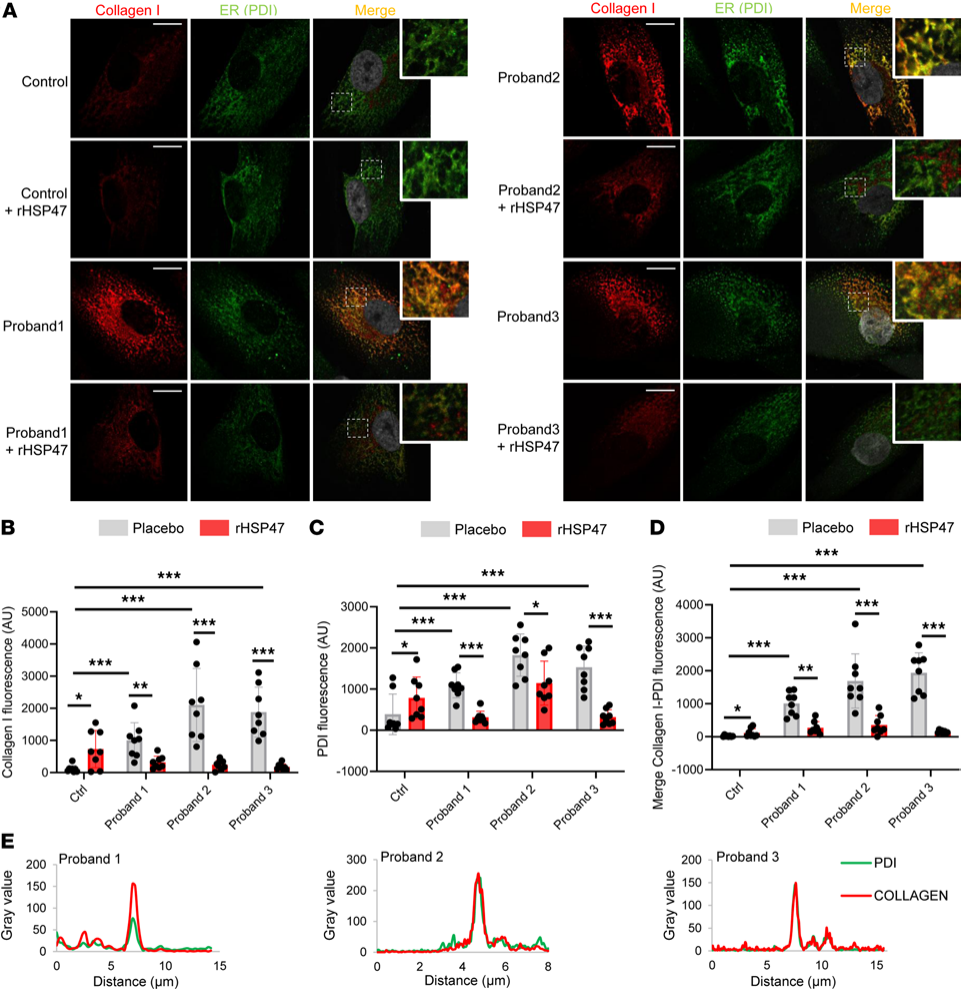
Treatment with rHSP47 reduces intracellular procollagen retention. (**A**–**D**) Representative images (**A**) and quantification of immunofluorescence of (**B**) collagen I (aligned rank ANOVA [ARA] test, *F*_ARA_ = 18.06, *P* < 0.001 by overall test), (**C**) ER marker protein disulfide isomerase (PDI) (ARA test, *F*_ARA_ = 15.12, *P* < 0.001 by overall test), and of (**D**) collagen I–PDI signal (ARA test, *F*_ARA_ = 40.35, *P* < 0.001 by overall test) of osteogenesis imperfecta (OI) proband and control fibroblasts treated for 16 hours with 0.5 μM rHSP47 or with placebo. Asterisks in **B**–**D** indicate the post hoc test results. Biological replicates (*n* = 3) were performed. For each biological replicate, the signal was quantified on 8 images (original magnification, ×40) for each genotype/condition (number of cells, >90). Error bars indicate SD. (**E**) The colocalization of collagen I–PDI signal in proband 1, 2, and 3 cells is evident by the 2 overlapping peaks in the graph (*n* = 3). Nuclei were counterstained with DAPI. Scale bars: 15 μm. Zoomed inset magnification, ×3. Statistical analyses details are reported in Supplemental Table 2. *P < 0.05; **P ≤ 0.01; ***P ≤ 0.001.

The therapeutic potential of rHSP47 was then evaluated in fibroblasts from patients with OI. Primary fibroblasts from 3 previously described recessive OI patients with mutations in components of the collagen 3-prolyl hydroxylation complex (32, 33) were selected for the study (Table 1). As expected based on previous data (31), a strong intracellular procollagen accumulation together with a higher level of the ER marker PDI were observed in type VII and VIII OI proband primary fibroblasts compared with controls (Figure 2, A–C). The abnormal collagen strongly colocalized with PDI (Figure 2, D and E). rHSP47 incubation significantly decreased the amount of intracellular collagen, to the level of control fibroblasts, in all mutant fibroblasts.

### rHSP47 ameliorates cell homeostasis in OI probands’ fibroblasts by increasing collagen I secretion.

**Figure 3:**
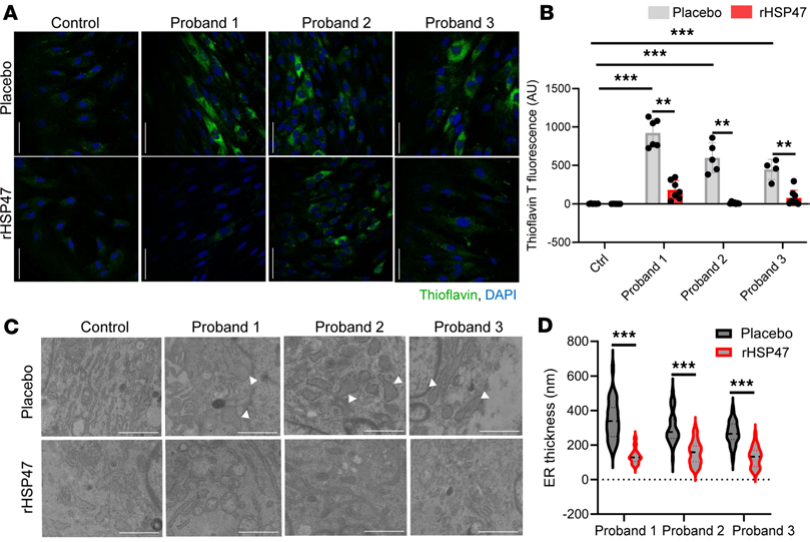
rHSP47 ameliorates cellular homeostasis. ER proteostasis was evaluated by thioflavin T (ThT) labeling of protein aggregates in osteogenesis imperfecta (OI) proband and control fibroblasts treated for 16 hours with 0.5 μM rHSP47 or with placebo. (**A**) Representative immunofluorescence images and (**B**) ThT quantification (aligned rank ANOVA [ARA] test, *F*_ARA_ = 86.82) is shown (*P* < 0.001 by overall test; asterisks indicate post hoc test results). Biological replicates (*n* = 3) were performed. For each biological replicate, the signal was quantified on 7 images (original magnification, ×40) for each genotype/condition (number of cells >70). Error bars indicate SD. Scale bars: 20 μm. (**C**) Representative transmission electron microscopy images of OI probands and control fibroblasts treated for 16 hours with 0.5 μM rHSP47 or with placebo (*n* = 3). Arrowheads show ER enlargement. Scale bars: 2 μm. (**D**) ER enlargement was quantified for 30 proband cells treated with rHSP47 or with placebo. Mann-Whitney Wilcoxon test, MW = 6.35 (*P* < 0.001 by overall test; asterisks indicate post hoc test results). Statistical analyses details are reported in Supplemental Table 2. **P ≤ 0.01; ***P ≤ 0.001.

**Figure 4:**
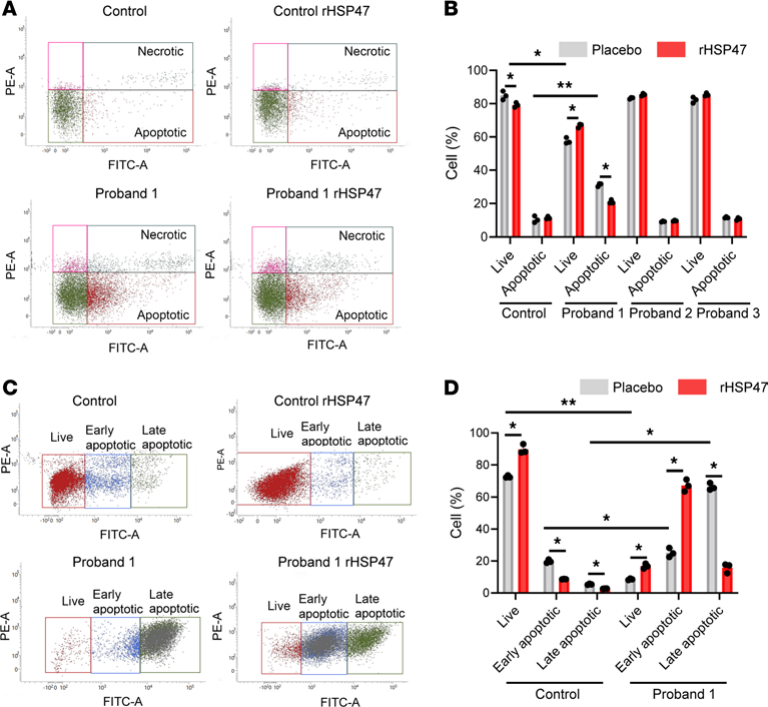
rHSP47 modulates cell apoptosis. (**A**) FACS analysis detection of apoptotic cells in OI probands and control fibroblasts treated with rHSP47 or with placebo following annexin V (FITC) and propidium iodide (PI) staining. The fraction of apoptotic events in the cells is shown in representative plots and (**B**) quantified in the histogram. Biological replicates (*n* = 3) were performed. Error bars indicate SD. (**C**) FACS analysis of apoptotic cells in OI proband 1 and control fibroblasts treated with rHSP47 or with placebo following a high-stress condition induced by culturing the cells for 7 days without media change. The fraction of apoptotic events in the cells is shown in representative plots and (**D**) quantified in the histogram. Both early and late apoptotic cells were quantified. Biological replicates (*n* = 3) were performed. Error bars indicate SD. Mann-Whitney Wilcoxon test was performed. Statistical analyses details are reported in Supplemental Table 2. *P < 0.05; **P ≤ 0.01.

The effect of rHSP47 was also evaluated on general ER proteostasis by treating cells with the protein aggregate–binding fluorescent molecule thioflavin T (ThT) (34, 35). Enhanced ThT fluorescence was found in all proband cells compared with controls, demonstrating the intracellular accumulation of misfolded material that was significantly reduced following rHSP47 treatment (Figure 3, A and B). The impact of rHSP47 on intracellular protein aggregates was also mirrored by an improvement in cellular morphology; the ER cisternae, enlarged in mutant cells, normalized after treatment (Figure 3, C and D). The impaired proteostasis severely affects vitality in OI cells, often inducing apoptosis activation (30, 31, 36–39). rHSP47 treatment decreased the number of apoptotic cells when an increased amount was present, as in proband 1, while it did not affect vitality in the other cases (Figure 4, A and B). Of relevance, upon the induction of a high-stress condition by culturing the cells for 7 days without media change, the positive effect of the treatment was even more pronounced on cell viability. Indeed, a strong decrease in the population of late apoptotic cells, and an increase in the percentage of early apoptotic and live cells was evident in mutant cells treated with rHSP47 (Figure 4, C and D).

**Figure 5:**
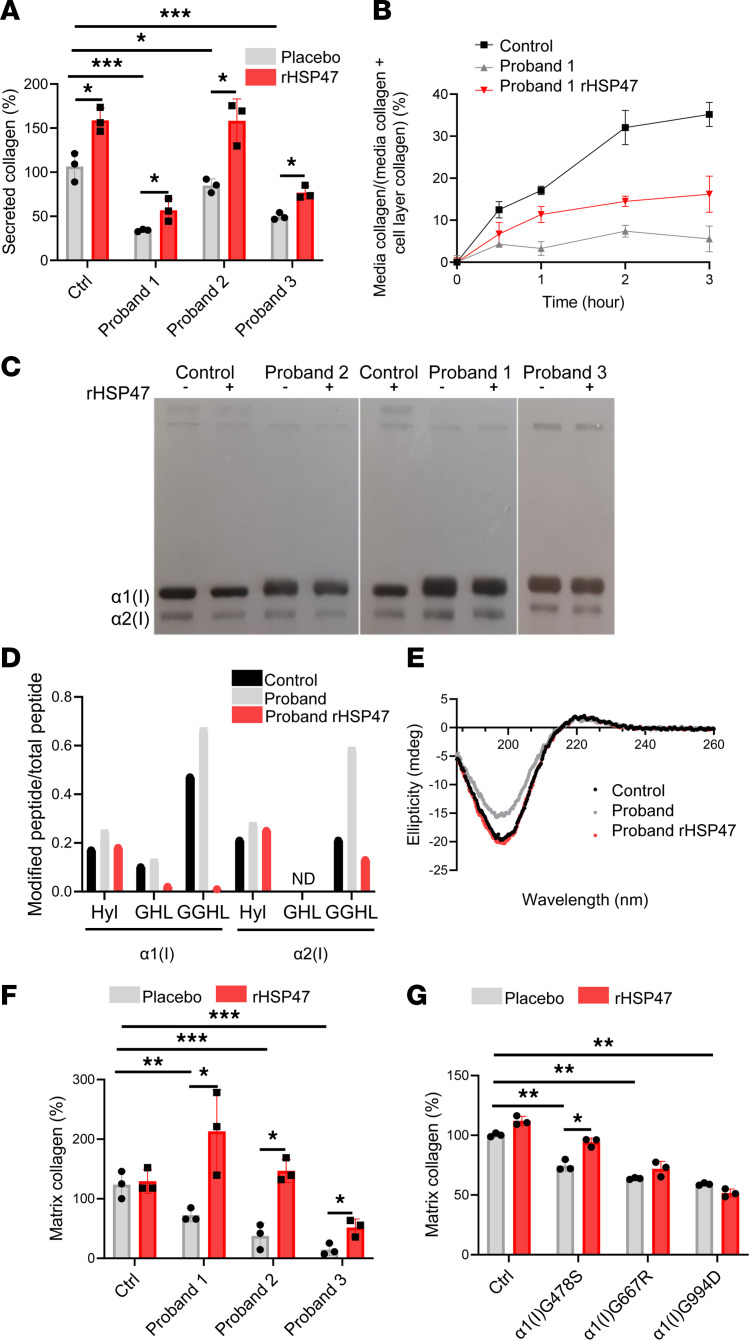
rHSP47 increases collagen secretion, reduces collagen overmodifications, and enhances collagen I deposition in the ECM. (**A**) rHSP47 effect on collagen secretion was evaluated in osteogenesis imperfecta (OI) probands and control fibroblasts. Secreted collagen was quantified in the last 24-hour culture media of fibroblasts after 7 days of culture with or without a 4-hour rHSP47 pulse (0.5 μM) every other day. Biological replicates (*n* = 3) were performed. For each biological replicate, collagen was quantified in 3 different wells for each condition. Kruskal-Wallis (KW) test, KW = 17.93 (*P* = 0.0005 by overall test; asterisks indicate post hoc test results). (**B**) Collagen secretion kinetics was evaluated in proband 1 by incubating the cells for 4 hours with ^3^H-proline. Technical replicates (*n* = 3) were performed. (**C**) Representative SDS-urea-PAGE of ^3^H-labeled collagen extracted from the medium of control and OI probands’ fibroblasts treated for 16 hours with 0.5 μM rHSP47 or with placebo. Biological replicates (*n* = 3) were performed. (**D**) Tandem mass spectrometry data of collagen I extracted from culture media of control and proband fibroblasts to evaluate lysyl hydroxylation and lysine O-glycosylation along the collagen helix (*n* = 3, pooled). Hydroxylysine (Hyl), galactosyl-hydroxylysine (GHL), and glucosylgalactosyl-hydroxylysine (GGHL) sites were identified by the analysis. The ratio between the posttranslationally modified peptides and the total peptides is reported. Light gray bars show proband 3 and red bars show proband 3 rHSP47. (**E**) Circular dichroism spectra reveal the collagen triple helical signal as a positive peak at 222 nm and negative peak below 200 nm in all samples (*n* = 3). Proband 3 is shown in light gray and proband 3 rHSP47 is shown in red. (**F**) The amount of collagen incorporated into the ECM was evaluated by Picrosirius red staining in cells grown for 7 days in the absence or presence of a 0.5 μM rHSP47 4-hour pulse performed every other day. Biological replicates (*n* = 3) were performed. For each biological replicate, collagen was quantified in 3 different wells for each condition. (**G**) The amount of collagen incorporated into the ECM was evaluated in fibroblasts from probands with collagen I mutations as reported in **F** (*n* = 3). Error bars indicate SD. Mann-Whitney Wilcoxon test was applied. Kruskal-Wallis (KW) test was also applied to describe the secreted collagen by genotype. Statistical analyses are described in Supplemental Table 2. *P < 0.05; **P ≤ 0.01; ***P ≤ 0.001.

The impairment of the collagen prolyl 3-hydroxylation complex in primary human fibroblasts decreases collagen I secretion, leading to a matrix insufficiency (31). Thus, secreted collagen I was quantified in culture media in the absence or presence of rHSP47 (Figure 5A). A significant increased amount of collagen content following rHSP47 incubation was evident in all the samples. ^3^H-labeled collagen I pulse-chase experiments performed in fibroblasts form proband 1 further confirmed the chaperone stimulatory effect on collagen I secretion (Figure 5B).

### rHSP47 improves the quality of the secreted collagen molecules and their presence in the matrix.

The impairment of the collagen prolyl 3-hydroxylation complex affects collagen I folding, causing increased collagen hydroxylation and glycosylation (14). In all analyzed OI proband cells, the presence of collagen overmodifications was confirmed by electrophoretic analysis of ^3^H-labeled collagen I. Steady-state collagen gels revealed the broadening and delay of the α bands typical of excessive glycosylated collagen I. rHSP47 incubation slightly reduced the α(I) band broadening, suggesting a reduced collagen PTM level (Figure 5C). To evaluate lysyl hydroxylation and lysine O-glycosylation along the collagen helix, collagen extracted from control, OI proband 3, and rHSP47-incubated OI proband 3 cells was subjected to trypsin digestion and liquid chromatography–tandem mass spectrometry (LC-MS/MS) analysis was performed. Several hydroxylysine (Hyl), galactosyl-hydroxylysine (GHL), and glucosylgalactosyl-hydroxylysine (GGHL) sites were identified by LC-MS/MS analysis: 4 Hyl (K^252^, K^270^, K^327^, and K^603^), 1 GHL (K^99^), and 4 GGHL sites (K^87^, K^99^, K^408^, and K^855^) detected in the α1 chain, and 4 Hyl (K^264^, K^270^, K^408^, and K^567^) and 4 GGHL sites (K^87^, K^264^, K^564^, and K^648^) detected in the α2 chain (Figure 5D, Table 2, and Supplemental Table 1; supplemental material available online with this article; https://doi.org/10.1172/jci.insight.181570DS1). The abundance of peptides containing Hyl and the abundance of GHL/GGHL peptides, as well as of the corresponding unmodified peptides, were calculated using Skyline software to compare the peak intensities across the specimen. The ratio between the peak intensity of modified peptides and the total intensity of all corresponding peptide forms (unmodified + hydroxylated + mono-O-glycosylated + di-O-glycosylated) indicated an increased O-glycosylation of both collagen α1(I) and α2(I) chains in OI proband cells. The incubation with rHSP47 significantly reduced collagen lysine hydroxylation and O-glycosylation at several residues. The lysine hydroxylation ratio was reduced at α1-K603 and at α2-K264 and -K270, the galactosylated ratio and galactosyl-glucosylated ratio were reduced at α1-K99 and at α1-K408, respectively, while α2-K264, -K564, and -K648 values were close to that of control (Table 2). No changes were detected at α1-K87 and α2-K87, relevant sites for collagen cross-links.

Collagen triple helical structure before and after treatment was assessed by circular dichroism (CD), monitoring collagen ellipticity at different wavelengths. An ellipticity change in the negative band at 200 nm, sensitive to triple helical structure (40), was evident in the CD spectra of collagen from proband 3 compared with control, supporting the abnormal structure of mutant collagen. Confirming a positive effect of HSP47 on collagen I structure, the mutant collagen ellipticity at 200 nm returned to control values upon rHSP47 treatment (Figure 5E).

To quantify the amount of collagen I incorporated into the ECM, upon decellularization, collagen staining was performed with Picrosirius red, a strong anionic dye that binds preferentially to the cationic groups of collagen. The spectroscopic quantification of the dye clearly revealed a significant increase in collagen in all probands’ ECM (Figure 5F). The collagen content in the ECM of proband 1 treated with rHSP47 was even greater than that of controls, underscoring the relevance of choosing a proper dose to avoid excessive collagen production and thus fibrosis in vivo.

The drug effect on collagen deposition was then evaluated, upon cell decellularization, also in fibroblasts from patients with the following collagen I mutations: α(I)G478S, α(I)G667R, and α(I)G994D. While we found a positive effect of rHSP47 in α(I)G478S fibroblasts with the most N-terminal mutation, no effect was found in α(I)G667R and α(I)G994D, cells with more C-terminal mutations (Figure 5G), suggesting that the effect of the treatment is likely mutation dependent.

### rHSP47 improves mineralization in zebrafish larvae.

**Figure 6:**
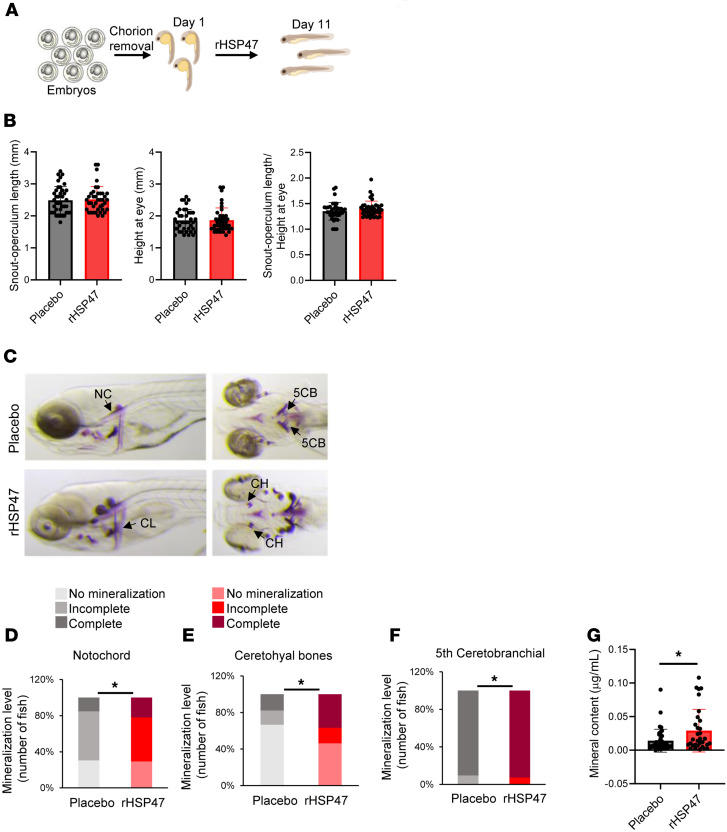
Treatment with rHSP47 ameliorates zebrafish *p3h1^–/–^* larvae bone mineralization. (**A**) Upon mechanical chorion removal, embryos from prolyl 3-hydroxylase 1–knockout (*p3h1^–/–^*) zebrafish were treated every other day with a 4-hour pulse of 0.5 μM rHSP47 or with placebo. At 11 dpf, the skeletons of larvae were stained with alizarin red in order to assess the rHSP47 effect on mineralization. (**B**) Analysis of the fish morphology was carried out measuring the snout-operculum length, the height at eye, and the snout-operculum length/height-at-eye ratio (*n* ≥ 40). (**C**) Lateral and ventral images of *p3h^–/–^* larvae, treated with rHSP47 or placebo, upon mineral staining by alizarin red. (**D**–**F**) Bone mineralization level analyzed in rHSP47-treated and untreated fish. Three classes of mineralization were defined, namely, no mineralization, incomplete, and complete, based on the level of alizarin red staining. Biological replicates (*n* ≥ 41) were performed. (**G**) Total larvae mineral content was extracted and quantified by spectrophotometric analyses. Biological replicates (*n* = 32) were performed. Error bars indicate SD. Mann-Whitney Wilcoxon test was applied. Statistical analyses details are reported in Supplemental Table 2. *P ≤ 0.05.

To investigate in vivo the effect of exogenous rHSP47 on the skeleton, we took advantage of the *p3h1^–/–^* zebrafish, recently generated and characterized in our laboratory as a valid model for recessive OI type VIII. Indeed, *p3h1^–/–^* larvae are characterized by head disproportion and by an impaired cranial bone mineralization starting from the first weeks after fertilization (41). From 1 to 11 days post fertilization (dpf), *p3h1^–/–^* embryos were treated every other day with a 4-hour pulse of 0.5 μM rHSP47 (Figure 6A). Analyses of the fish morphology at 11 dpf revealed no effect on the head disproportion since the snout-operculum length/height-at-eye ratio was unaffected by the treatment (Figure 6B). At 11 dpf, bone mineralization was also evaluated by alizarin red S staining. Three classes of mineralization were defined, namely, beginning/no mineralization, incomplete, and complete, based on the level of alizarin red staining. The staining revealed a significant increased mineralization of notochord (NC), ceratohyal (CH), and fifth ceratobranchial (5CB) (Figure 6, C–F), cranial bone characterized by perichondral and endochondral ossification, respectively. Furthermore, the spectrophotometric quantitation of the total larval mineral content confirmed the significantly increased mineralization upon rHSP47 incubation (Figure 6G).

## Discussion

The reported data provide the proof of principle for exogenous rHSP47 administration as promising treatment for diseases characterized by poor collagen I secretion and matrix incorporation. Both in vitro and in vivo approaches using OI type VII (33, 42) and VIII (43) human fibroblasts and a zebrafish OI type VIII model (*p3h1*^−/−^) (41), respectively, support our conclusions. Mutant fibroblast incubation with rHSP47 reduced collagen PTMs, increased collagen secretion and matrix incorporation, and ameliorated the general ER proteostasis, leading to an improved cellular homeostasis and vitality. These positive cellular effects translated in vivo to a significantly improved bone mineralization. Of note, a mutation-dependent effect was found in fibroblasts from probands with collagen I mutations where the treatment was effective only in cells with the most N-terminal mutation.

Collagen I retention and its only partial secretion from the ER causing a defective ECM deposition are common features in dominant OI types carrying mutations in collagen I genes and in several recessive OI forms characterized by collagen structural defects (31, 36–38, 44–46). Furthermore, reduced collagen synthesis and matrix insufficiency are hallmarks of aging-related skeletal diseases such as osteoporosis, thus representing appealing pharmacological targets to ameliorate human health and reduce health care system expenses in an aged society. Despite that, the search for therapies addressing these targets is still exiguous. The anti-sclerostin antibody, the only currently available drug approved for osteoporosis and in clinical trials for OI that enhances collagen deposition, does not specifically target collagen but, being an inhibitor of the WNT pathway, exerts an effect on other essential cellular functions, with potential serious side effects (47, 48). Similarly, lack of collagen specificity characterizes the most recent anti–TGF-β antibody treatment that is in clinical trials as an inhibitor of the tumor microenviroment and metastasis and as a bone mineral density inducer for OI (49). In the last decade, in vitro and in vivo preclinical studies by us and others identified in the chemical chaperone 4-phenylbutyrate (4-PBA) a new anabolic drug able to ameliorate OI osteoblast homeostasis and skeletal properties both in a zebrafish and in 2 murine models of dominant OI (50–52). In vitro studies using Chinese hamster ovary cells revealed that 4-PBA promotes proper COP2 coat assembly, known to be necessary for large ER proteins’, including collagen I molecules, transit from the ER to the Golgi (53). Nevertheless, 4-PBA, similarly to the above-mentioned anabolic drugs in clinics or clinical trials, is not specific for collagen and does not affect the OI-altered collagen structure.

The chemical chaperone HSP47 specifically interacts with collagen I, facilitating its folding, limiting its intracellular aggregation, and allowing its secretion. Thus, taking this information into account, we reasoned that HSP47 administration could fill the lack of drugs specifically targeting collagen I synthesis, modulating its PTM levels and favoring its secretion (28, 54). The expression of HSP47 correlates with collagen deposition (28). On the other hand, knocking out HSP47 expression in mouse embryos results in abnormally oriented epithelial tissues and ruptured blood vessels due to severe deficiency in mature forms of type I and type IV collagen leading to a lethal phenotype (55), while in humans homozygosity for a missense mutation causes a recessive moderate to severe form of OI with distinct effects on collagen I secretion and structure depending on the type of defect (56).

While rHSP47 administration to healthy individuals may be detrimental because elevated expression of HSP47 is observed in a variety of fibrosis models (57) and has been associated with the promotion of cancer (58), administration to individuals with impaired collagen secretion would potentially help to overcome matrix insufficiency.

Our data demonstrated the cellular uptake of an exogenously provided recombinant form of HSP47 in human fibroblasts, providing information regarding its localization. The HSP47 RDEL C-terminal motif is known to bind to the KDEL receptor (KDELR) in the ER-Golgi intermediate compartment, but KDELRs are also present in the plasma membrane (PM) (59). Following ligand interaction, the PM-localized KDELR1 was shown to be endocytosed into early endosomes via clathrin-mediated endocytosis before entering the Golgi and then recycled back to the endosomal compartment or to the PM (59). The rHSP47-GFP colocalization with COP2, KLHL12, and GM130 by immunofluorescence demonstrated its presence at the ER exit site (ERES). ERESs, specific exit points for ER-synthesized proteins, are specialized membrane domains on the surface of the ER that are identified by the COP2 heterocomplex and ER-Golgi recycling proteins such as ERGIC53 (60). The procollagen molecules are recruited to the ERES by the ERES resident protein TANGO1, whose SH3 domain is bound by HSP47 interacting with a procollagen triple helix (28, 61). A recent model for procollagen secretion proposed an active role of proton and HSP47 concentration gradients existing between ERES and *cis*-Golgi in generating the necessary biomechanical driving force to allow procollagen through tunnels present between the ER and Golgi (5). The localization of rHSP47 at the ERES site could indeed explain a higher concentration gradient possibly being responsible for the increased rate of collagen secretion detected in treated mutant fibroblasts. HSP47 is known to preferentially bind to folded triple helical procollagen, stabilizing an otherwise thermally unstable molecule, whereas PTMs are reported to occur on the unfolded collagen chains and to be blocked from their winding (62). Thus, quite unexpectedly, the exogenous rHSP47 decreased collagen hydroxylation and glycosylation, as demonstrated by MS data. An intriguing explanation could be a feedback effect of the increased presence of exogenous rHSP47 on the transcription/translation of other PTM enzymes and chaperones. It has been recently reported that, in a recessive OI patient carrying a mutant HSP47-R222S, collagen I was overglycosylated, likely due to an increased expression of chaperones and modifiers compensating for the abnormal HSP47 collagen I binding (63). The authors hypothesized that presumably those proteins were occupying the vacant HSP47 binding sites, causing collagen overmodification. Another possible explanation could be the existence of relevant collagen PTMs in the ER-to-Golgi transition tunnel where the triple helices are already assembled, but where HSP47 is starting to be released due to lowering of the pH. Indeed, a post-Golgi trafficking mediated by VIPAR that is essential for modification of lysine residues by lysyl hydroxylase 3 (LH3) in multiple collagen types has been discovered, including type I (64). Thus, it is possible that the faster collagen secretion upon HSP47 treatment also reduces the time for LH3 collagen modifications.

The rHSP47 incubation is particularly promising for OI treatment since it specifically targeted at least 3 hallmarks of OI, namely, it reduces PTMs, it increases collagen synthesis and thus limits intracellular retention, and it increases the collagen amount in the ECM. Nevertheless, our findings also revealed the need for optimizing dose and time of treatment. For instance, the collagen I content in the ECM of proband 1 treated with rHSP47 was even greater than the collagen amount of controls, as well as the levels of GHL and GGHL in α1(I) upon treatment are reduced to levels even lower than that of control, underscoring the relevance of the choice of a proper dose to avoid in vivo an excessive collagen production and thus fibrosis or the synthesis of undermodified collagen molecules. Of relevance, collagen undermodification, a peculiar feature found in patients with OI type XIV, is detrimental for bone properties as well as its overmodification.

The efficacy of the treatment was here first tested in probands with mutations impairing the collagen prolyl 3-hydroxylation complex and causing the synthesis of collagen molecules with similar levels of PTMs. OI types caused by mutations in the collagen prolyl 3-hydroxylation complex, and in a few other genes encoding proteins involved in collagen folding and PTMs (i.e. *FKBP10*, *MESD*, and *PLOD*), are the more common among the recessive OI forms, representing more than 880 individuals (65). Nevertheless, the findings that the effect of the treatment is likely mutation dependent in case of collagen I mutations, and that, perhaps for patients with more C-terminal mutations and higher PTM levels, the effective HSP47 dose could be different or the treatment could be less effective, represent a potential limit of the proposed treatment. Indeed, the position of the mutation, the type of substituting amino acid, and the affected chain result in collagen with heterogeneous modifications, and this could be responsible for the different response. Furthermore, the translatability of these promising positive effects from fibroblasts to osteoblasts, which are known to synthesize higher collagen amount, is still pending. For instance, working with induced pluripotent stem cells isolated from patients with OI and differentiated toward osteoblasts could provide additional useful insights.

Besides bone and skin, collagen I is present in the connective tissues of many other organs, providing the tissues with tensile strength. In particular, it is present in all major structures of the lung, in the skeletal muscle where it accounts for 1%–10% of its mass dry weight and it plays an important role in muscle fiber force transmission, and it is a major component of cardiac valves and the aortic wall and its altered synthesis can affect their biomechanical properties (66), leading, in rare cases, to aortic dissection (67). The use of exogenous rHSP47 as OI treatment, unlike other proposed drugs, could target all OI-affected tissues, likely also improving the extraskeletal manifestations of the diseases, a major concern for adult patients (68). Of relevance, with HSP47 being a specific chaperone for fibrillar collagen, the same therapeutic approach could be applied to several other conditions in which collagen synthesis is impaired, either genetically or acquired due to environmental factors, such as epidermolysis bullosa (69), Ehlers-Danlos syndrome (15), chondrodysplasia (70, 71), UV exposure (72), and smoking (73–75).

Our study demonstrated the power of exogenous rHSP47 in ameliorating cellular homeostasis and bone mineralization using in vitro and in vivo models of recessive OI forms characterized by collagen posttranslational overmodifications. The unchanged disproportion in *p3h1^–/–^* treated larvae underscores the need to evaluate the effect of a long-term treatment, for which a specific delivery system for the recombinant protein is necessary.

The identification of specific therapies for the different OI forms, or even for a specific group of mutations in the same gene, is the seed for the development of a personalized medicine approach, with the final aim to ameliorate OI-affected individuals’ quality of life.

Although the analysis of rHSP47’s effect on bone fragility and the potential side effects of a high HSP47 level require extensive in vivo investigation, our results pave the way for a new pharmacological approach for OI, as well as other rare and common disorders associated with collagen deficiency.

## Methods

### Sex as a biological variable

Our study examined skin biopsies from male (*n* = 1) and female (*n* = 2) individuals with OI and individuals without disease acting as controls. Sex was not considered as a biological variable. For zebrafish embryonic/juvenile experiments, clutch-matched fish were randomly assigned to each treatment group and used without sex bias.

### rHSP47 expression and purification

Recombinant HSP47 protein (rHSP47), nonconjugated and conjugated with EGFP, was kindly provided by Syuzanna Hambardzumyan and by Essak Khan (INM-Leibniz Institute for New Materials, INM, Campus D2 2, Saarbrück, Germany). A construct encoding amino acids 36–418 of canine HSP47 (canine *SERPINH1* mRNA, NCBI accession NM_001165888) was cloned into the pET22-(b) vector (Novagen) with a C-terminal hexahistidine tag. Proteins were expressed in BL21(DE3) cells and purified by Ni-NTA affinity chromatography (Ni-NTA superflow; Qiagen). The eluate was reduced with 4 mM DTT, and 1.5 M ammonium sulphate was added to precipitate contaminants. The soluble fraction was concentrated and purified further by gel filtration (Superdex S75; GE Healthcare) in 20 mM Hepes pH 7.5, 300 mM NaCl, 4 mM DTT.

### Human fibroblast culture and rHSP47 treatment

Human primary dermal fibroblasts from skin biopsies of pediatric OI probands carrying mutations in one of the genes coding for the members of the collagen prolyl 3-hydroxylation complex, *CRTAP* (OI type VII; refs. 33, 42) or *P3H1* (OI type VIII; ref. 43) (Table 1), and in collagen I α1 chain [α1(I)G478S, α1(I)G667R, and α1(I)G994D] (76), were obtained after written informed consent. All the primary cells were isolated and grown at the department of Molecular Medicine of the University of Pavia, Italy, with the exception of *CRTAP*-mutant cells that were isolated at the Department of Physiology and Cell Biology of the University of Arkansas for Medical Sciences, Little Rock, Arkansas, USA. Three aged, matched controls (Promo Cell) were selected. All cells were used at passages 7–12. Cells were grown at 37°C in humidified atmosphere containing 5% CO_2_ and cultured in Dulbecco’s modified Eagle’s medium (DMEM, Lonza) supplemented with 10% fetal bovine serum (FBS, Euroclone) and 4 mM glutamine (Euroclone). For each experiment, 2.5 × 10^4^ cells/cm² were plated and cultured in DMEM with 10% FBS and 50 μg/mL ascorbic acid. Cells were harvested after 5 days. For treatment, cells were incubated with 0.5 μM rHSP47 (dissolved in PBS) or with placebo (same volume of PBS) for 16 hours with the exception of collagen secretion and matrix incorporation experiments, for which cells were subjected to a 4-hour pulse incubation with rHSP47/placebo every other day. After each treatment, cells were washed 3 times with PBS and fresh medium was added. For HSP47 localization, cells were incubated with 0.5 μM rHSP47-GFP (dissolved in PBS) for 4 hours.

### Immunofluorescence for intracellular localization

#### Collagen localization.

Human primary fibroblasts were plated on glass coverslips (Marienfeld) and after 24 hours treated with 0.5 μM rHSP47 for 16 hours. For collagen localization studies, cells were fixed in 10% neutral buffered formalin. For collagen colocalization with the ER marker PDI and with the Golgi marker GM130, cells were sequentially incubated overnight with antibodies against collagen I α1 chain (Developmental Studies Hybridoma Bank, SP1.D8; 1:100), PDI (Cell Signaling Technology, 3501S; 1:200), and GM130 (Alexa Fluor 647; BD Pharmingen, 558712; 1:100). The secondary antibodies Alexa Fluor 546–goat anti–mouse IgG (Invitrogen, A-11030) and Alexa Fluor 488–conjugated F(ab′) fragment anti–rabbit IgG (Immunological Sciences, IS20013) were used at 1:400 dilution. Nuclei were stained with 4′,6-diamidino-2-phenylindole (DAPI, Sigma-Aldrich) and images were acquired by confocal microscope TCS SP8 (Leica). The total area of punctate signal per cell and signal colocalization were measured using Leica software LAS 4.5.

#### rHSP47 localization.

Human primary fibroblasts were plated on glass coverslips and after 24 hours treated with 0.5 μM rHSP47-GFP for 16 hours. In order to evaluate rHSP47-GFP colocalization with the endoplasmatic reticulum (ER), immunofluorescence experiments for the ER matrix marker PDI and ER membrane marker calnexin were performed. After cell fixation with formalin, cells were blocked with 1.5% bovine serum albumin (BSA) and 0.3% Triton X-100 in PBS for 1 hour at room temperature (RT). Antibody against PDI (Cell Signaling Technology, 3501S) or against calnexin (Cell Signaling Technology, 2679) was incubated overnight at 4°C using a 1:200 dilution in blocking solution. Cells were sequentially incubated with goat anti–rabbit IgG DyLight 633 secondary antibody (Thermo Fisher Scientific, 35562) at 1:400 dilution in blocking solution, for 2 hours at RT.

To evaluate rHSP47-GFP colocalization with the Golgi apparatus, immunofluorescence for GM130 was performed. Cells were blocked with 0.5% BSA, 0.05% saponin, 50 mM NaCl, and 15 mM glycine, pH 7.4, for 1 hour at RT. Alexa Fluor 647–conjugated anti-GM130 (BD Pharmingen, 558712) was used at 1:100 dilution in blocking solution and incubated overnight at 4°C.

To evaluate rHSP47-GFP colocalization with secretory vesicles, immunofluorescent staining for COP2 and KLHL12 was performed. For COP2 immunodetection, cells were permeabilized with 0.1% Triton X-100 dissolved in TBS and blocked with 3% BSA for 1 hour at RT. Antibody against COP2 (Invitrogen, PA1-069) was used at 1:200 dilution in blocking solution and incubated overnight at 4°C. Cells were sequentially incubated with goat anti–rabbit IgG DyLight 633 secondary antibody (Thermo Fisher Scientific, 35562) at 1:400 dilution in blocking solution, for 2 hours at RT.

For KLHL12 immunodetection, cells were blocked with 0.5% BSA, 0.05% saponin, 50 mM NaCl, and 15 mM glycine, pH 7.4, for 1 hour at RT. Antibody against KLHL12 (Sigma-Aldrich Atlas Antibodies, HPA07132) was used at 1:400 dilution in blocking solution and incubated overnight at 4°C. Cells were sequentially incubated with goat anti–rabbit IgG DyLight 633 antibody (Thermo Fisher Scientific, 35562) at 1:400 dilution in blocking solution, for 2 hours at RT. Nuclei were counterstained with DAPI and images acquired by confocal microscope TCS SP8 (Leica) at ×63 magnification. The colocalization analyses were performed with ImageJ software (NIH).

### ThT labeling for protein aggregate analysis

Cells were plated on glass coverslips and cultured for 4 days before incubation with 5 mM ThT (Sigma-Aldrich) for 16 hours in the presence or absence of rHSP47. Cells were then fixed with 4% paraformaldehyde (PFA) and nuclei were stained with DAPI. Images were acquired by confocal microscope TCS SP8 (Leica). The total area of punctate signal per cell was measured using the Leica software LAS 4.5.

### Transmission electron microscopy to evaluate ER morphology and cisternae size

For transmission electron microscopy analysis, fibroblasts were trypsinized, fixed in 2.5% glutaraldehyde for 2 hours at RT, postfixed in 2% (w/v) OsO_4_ for 2 hours at RT, and embedded in 2% agarose. The specimens were dehydrated in graded acetone, infiltrated with epoxy resin, and finally polymerized in gelatin capsules. Ultrathin sections were cut on a Reichert OM-U3 ultramicrotome, collected on 300-mesh nickel grids, and stained with saturated aqueous uranyl acetate and lead citrate. A JEM 1200 EX II electron microscope operated at 100 kV and equipped with a MegaView G2 CCD camera was used for acquisition. ER cisternae thickness was measured with the built-in software on 30 cells per condition.

### Fluorescence-activated cell sorting assay for cell viability and apoptosis evaluation

To determine rHSP47’s effects on cell viability and apoptosis, cells were stained with annexin V–FITC conjugate and propidium iodide (PI) using a FITC annexin V/dead cell apoptosis kit (Invitrogen, V13242). Samples were analyzed by fluorescence-activated cell sorting (FACS) (BD FACS Lyric, Becton Dickinson). A total of 1 × 10^4^ events for each sample were analyzed, measuring the fluorescence emission at 515–545 nm (FITC) and 675–715 nm (PI), to avoid fluorescence spillover. The BD FACS Suite (v1.3) software supplied by the manufacturer was used for the analysis.

### Collagen quantification from cell media and ECM

Cells were cultured for 7 days and incubated with 0.5 μM rHSP47 for 4 hours every other day. On day 7, 24-hour media were collected and, following PBS washing, the matrix was decellularized by incubation for 10 minutes in 50 mM Tris-HCl, pH 8.0 containing 2 M KCl and 0.2% Triton X-100. The decellularized matrix was extensively washed with 10 mM Tris-HCl, pH 8.0. DNA extraction was performed to confirm decellularization. Collagen extraction and quantification from media and decellularized matrix was performed using a Sircol Soluble Collagen Assay (Biocolor).

### Collagen steady state and chase analysis

Labeling of collagen with L-[2,3,4,5-^3^H]-proline (PerkinElmer) was used to evaluate collagen overmodification and collagen secretion kinetics. Fibroblasts (2.5 × 10^5^ cells) were plated in 6-well plates and grown for 24 hours. Cells were then incubated for 2 hours with serum-free DMEM containing 100 μg/mL ascorbic acid (Fluka) to stimulate collagen production (prelabeling medium). For steady-state experiments, the labeling lasted for 18 hours in the same media using 20 μCi of ^3^H-Pro/well.

For chase experiments, the labeling was performed for 4 hours using 1.65 μCi of ^3^H-Pro/mL, and then the labeling media were replaced with serum-free DMEM containing 2 mM proline (Sigma-Aldrich), 4 mM glutamine, 100 μg/mL penicillin and streptomycin, and 100 μg/mL ascorbic acid (chase media). Collagen was collected at 0.5, 1, 2, and 3 hours after the pulse. Collagen I from both medium and cell layer fractions was extracted as previously described (77). The radioactivity (counts per minute, CPM) was measured using a liquid scintillation counter (Tri-Carb 2300 TR). For steady state, the same amount (CPM) of ^3^H-labeled collagen from each sample was denatured and run in 6% SDS-urea-PAGE gels. For chase analyses, the same volume of ^3^H-labeled collagens from each time point was electrophoresed. The gels were fixed in 45% methanol, 9% glacial acetic acid, incubated for 1 hour with enhancer (PerkinElmer), and washed in deionized water. Gels were dried and placed in contact with a radiography film at –80°C. Films were developed, and the α(I) band intensity was evaluated using ImageQuant TL analysis software (GE Healthcare). For chase analyses, the ratio between the collagen in the media and the total collagen (medium plus cell layer) was evaluated.

### MS analysis of collagen I PTMs

Nano-LC‑MS/MS analysis was used to assess hydroxylation and O-glycosylation of lysine sites of collagen I. Collagen I was extracted, as reported above, from culture media of control, OI proband 3, and rHSP47-treated OI proband 3 primary fibroblasts incubated overnight with 50 μg/mL ascorbic acid to stimulate collagen secretion. Media from 3 T25 confluent flasks for each condition were collected every other day for 3 times and pulled. Collagen was quantified, separated by SDS-PAGE, and stained with colloidal Coomassie; the α1(I) and α2(I) bands were excised and destained in 0.1% trifluoroacetic acid (TFA)/acetonitrile (ACN) 1:1 (v/v) (78). Each band was reduced, alkylated, and digested overnight with trypsin, sequencing grade (Promega, T7575) at 37°C using a protease/protein ratio of 1:10 (79). Proteolytic digests were extracted with 50% ACN in 0.1% TFA and desalted using μZip-Tip C18 (Pierce, 87784) before MS analysis (80). Nano-HPLC coupled to MS/MS analysis was performed on a Dionex Ultimate 3000 HPLC system with an EASY-Spray 2 μm 15 cm × 150 μm capillary column filled with 2-μm C18 100 Å particles, connected to a Q-Exactive Orbitrap (Thermo Fisher Scientific). MS spectra were collected over an *m*/*z* range of 350–2000 Da at a resolution of 70,000, operating in data-dependent mode. HCD MS/MS spectra were collected at a resolution of 17,500 for the 10 most abundant ions in each MS scan, using a normalized collision energy of 35% and an isolation window of 3 *m*/*z*. Rejection of +1 and unassigned charge states was enabled.

The acquired MS/MS spectra were searched against the UniProtKB/Swiss-Prot type I Collagen sequence database (release 2023_10) for *Homo* *sapiens* using Skyline software v23.1 (https://skyline.ms/project/home/software/Skyline/begin.view). Search parameters included digestion by trypsin with a maximum of 4 missed cleavage sites, a minimum peptide length of 7, a mass deviation of 10 ppm for monoisotopic precursor ions and 0.5 Da for MS/MS peaks. Carbamidomethylcysteine (+57.0236) was set as a fixed modification, while Met oxidation (+15.994916), Lys hydroxylation (Hyl +15.994916), Lys galactosyl hydroxylation (GHL +178.047738), and glucosyl galactosyl hydroxylation (GGHL +340.100562) were set as variable modifications. The peak heights of the monoisotopic extracted ion chromatograms of the Hyl and GHL/GGHL peptides of type I collagen [α1(I) and α2(I)] from control, OI proband 3, and rHSP47-treated OI proband 3 fibroblasts were compared, as well as the peak heights of the corresponding unmodified peptides. The ratio between the peak intensity of the modified peptides and the total intensity of all peptide’s forms (unmodified + hydroxylated + mono-O-glycosylated + di-O-glycosylated) was calculated.

### Collagen CD to analyse triple helical structure

Collagen was extracted from culture media of control, OI proband 3, and rHSP47-treated OI proband 3 primary fibroblasts incubated overnight with 50 μg/mL ascorbic acid to stimulate collagen secretion. Collagen I was precipitated with a half volume of 96% ethanol and digested overnight at 4°C with 100 μg/mL pepsin in 0.5 M acetic acid. The collagen was then precipitated with 2 M NaCl in 0.5 M acetic acid and resuspended in 0.02 M acetic acid. All collagen I samples were prepared to a final concentration of 0.05 μg/μL. CD spectra were acquired using a J-1500 spectrophotometer (Jasco). The measurements were performed at 4°C, evaluating ellipticity as a function of wavelength in a range between 185 nm and 260 nm.

### Alizarin red staining of zebrafish skeleton

WT AB zebrafish were obtained by European Zebrafish Research Center (Germany). *p3h1^–/–^* fish were generated in our laboratory (41) and bred in-house at the animal facility Centro di Servizio per la Gestione Unificata delle Attività di Stabulazione e di Radiobiologia of the University of Pavia, Pavia, Italy.

Adult zebrafish (AB) were kept in a ZebTec (Tecniplast) semiclosed recirculation housing system at 28°C, conductivity 500 μS, pH 7.5, and 14:10 hours light/dark cycle in the centralized animal facility of the University of Pavia. Zebrafish embryos were collected in 1.2 mM NaHCO_3_, 0.1 g/L Instant Ocean, 1.4 mM CaSO_4_, 0.00002% (w/v) methylene blue, and kept at 28°C in an incubator. Zebrafish *p3h1^–/–^* embryos were obtained by mating *p3h1^–/–^* fish.

Upon mechanical chorion removal, embryos were treated every other day with 0.5 μM rHSP47 (dissolved in PBS) or placebo (same volume of PBS) for 4 hours. Following the treatment, the fish water was changed with fresh water. The skeletons of 11 dpf larvae untreated (*n* ≥ 41) and treated (*n* ≥ 42) were stained as previously described with alizarin red S in order to assess the rHSP47 effect on mineralization (41). Images were acquired using a Leica M165 FC microscope connected to a Leica DFC425 C digital camera. Analyses of the fish morphology were carried out measuring the snout-operculum length, the height at eye, and the ratio snout-operculum length/height at eye. The level of ossification of NC, CH, and 5CB was qualitatively described from beginning/incomplete to complete ossification based on the intensity of the staining from 3 independent operators blinded about the treatment groups. Total alizarin was also quantified by spectroscopic detection at 405 nm following dissolution in 10% acetic acid at 85°C for 10 minutes. Alizarin red standards from 20 μM to 200 μM were used. Using lateral images, snout-operculum length, defined as the distance from snout to the most posterior point of operculum and height at eye, defined as the distance from ventral to dorsal, immediately posteriorly to the eye and perpendicularly to the body axis, were measured.

### Study approval

Zebrafish studies were approved by the Italian Animal Research Council under Protocol 260/2020-PR.

### Statistics

The aim of the present study was to describe the effect of genotype and rHSP47 treatment on several cellular and morphological parameters. An exploratory study design in vitro and in vivo using a zebrafish OI model was applied. The quantitative variables are expressed as mean and standard deviation (SD). Differences between rHSP47 treatment and placebo in cellular and morphological quantitative as well as pseudoquantitative parameters were explored by Mann-Whitney Wilcoxon test (MW test). Aligned rank ANOVA (ARA) equivalent to 2-way ANOVA was applied to explore differences in collagen I, PDI, collagen I–PDI, and ThT by treatment and genotype. Post hoc analysis of the genotypes with respect to controls was conducted if nonparametric ARA was significant; Bonferroni’s multiple-comparison correction was applied. To describe the effect of rHSP47 and genotype on secreted collagen, matrix collagen, and vital parameters, the analyses were conducted using an MW test stratifying by genotype and comparing rHSP47 treatment and placebo due to the small number of experimental units. The Kruskal-Wallis test was also applied to describe the secreted collagen by genotype. A *P* value of less than 0.05 was considered significant, apart from the multiple-comparison test. All the analyses were conducted in STATA 17 and are summarized in Supplemental Table 2.

### Data availability

Values for all data points in graphs are reported in the Supporting Data Values file.

The MS proteomics data have been deposited to the ProteomeXchange Consortium via the PRIDE partner repository with the data set identifier PXD047320.

## Author contributions

RB and AF designed the experiments. RB, NG, and AS performed experiments and analysed the results (NG and AS equally contributed to both experiments and analyses). FT, CA, EM, and CC performed specific experiments. GT and MB analysed specific results. SV and CT performed statistical analyses. RB and AF wrote the manuscript with helpful revision by AR, GT, AL, and EM. All the authors read and approved the manuscript.

## Supplementary Material

Unedited blot and gel images

Supplemental table 1

Supplemental table 2

Supporting data values

## Figures and Tables

**Table 1:**
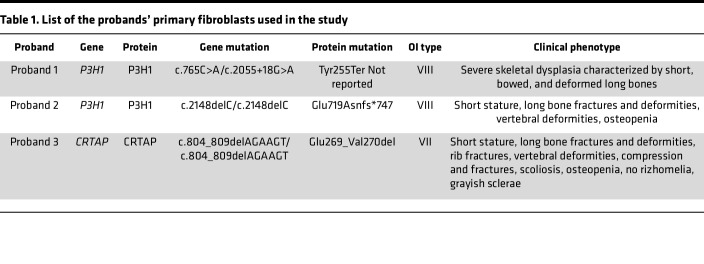
List of the probands’ primary fibroblasts used in the study

**Table 2:**
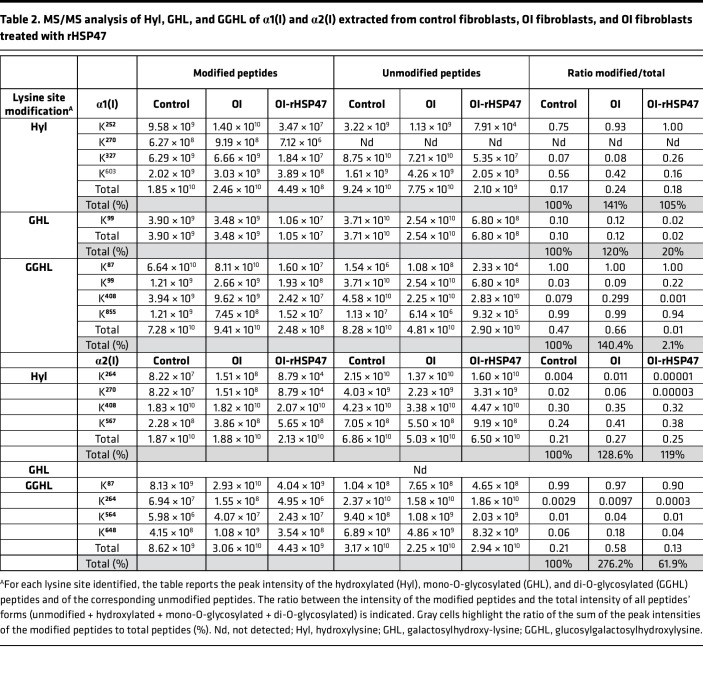
MS/MS analysis of Hyl, GHL, and GGHL of α1(I) and α2(I) extracted from control fibroblasts, OI fibroblasts, and OI fibroblasts treated with rHSP47

## Version 1 02/06/2025

In-Press Preview

## Version 2 03/24/2025

Electronic publication
