# Supplementary figures and images for: The administration of exogenous HSP47 as a collagen-specific therapeutic approach
Source: JCI Insight. 2025 Feb 6;10(6):e181570. doi: 10.1172/jci.insight.181570 (PMC11949040; doi:10.1172/jci.insight.181570)

Row images figure 5c

Final image

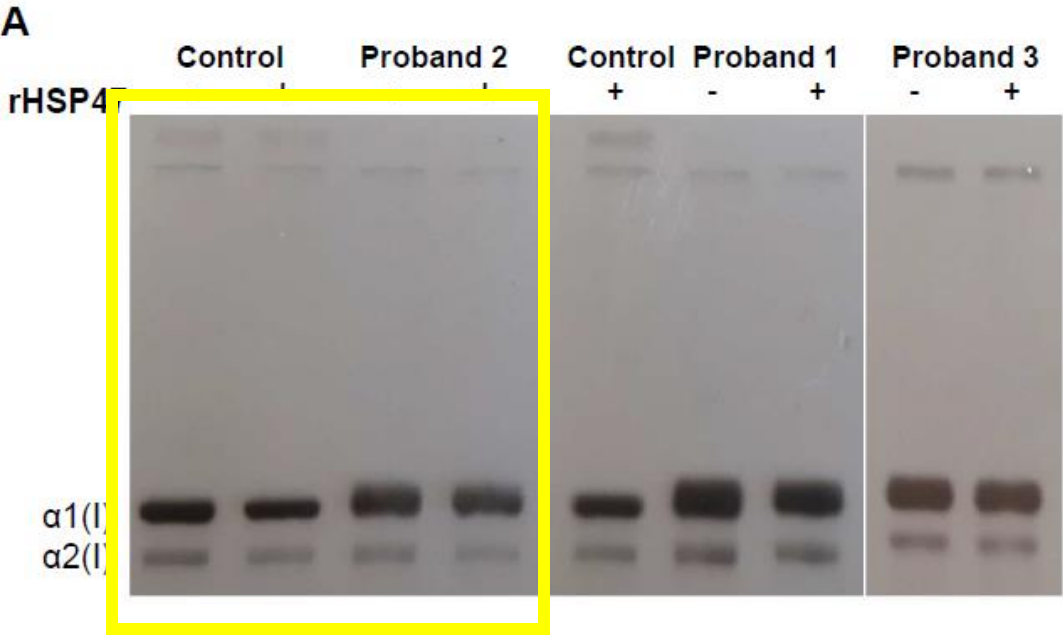

Row image

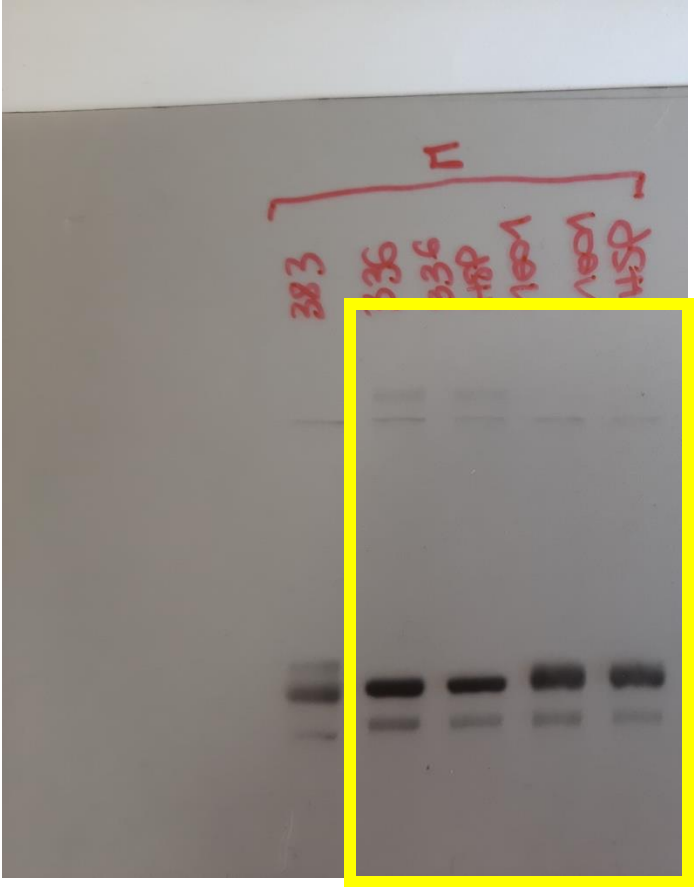

### Row image

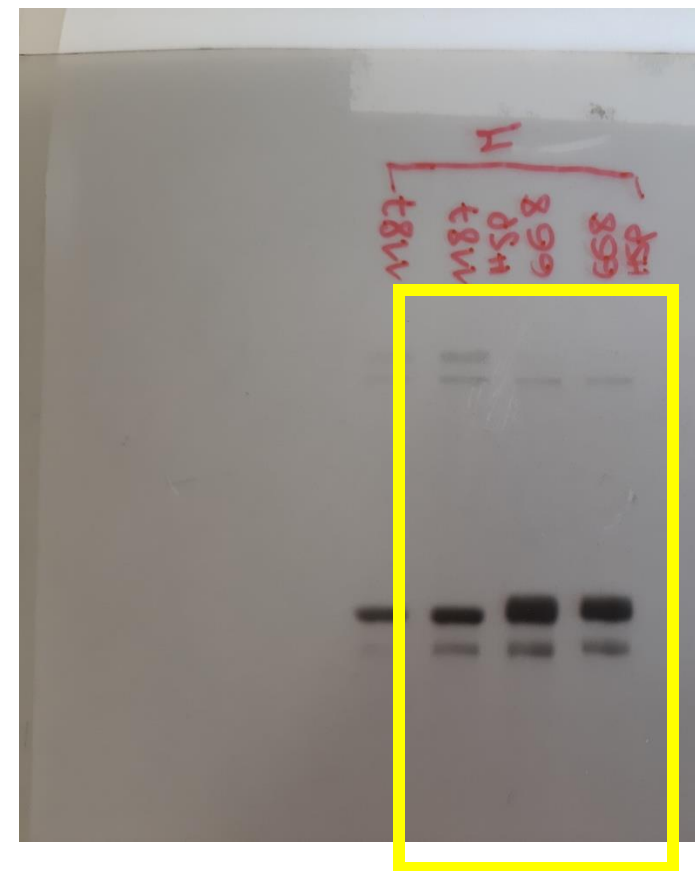

Final image

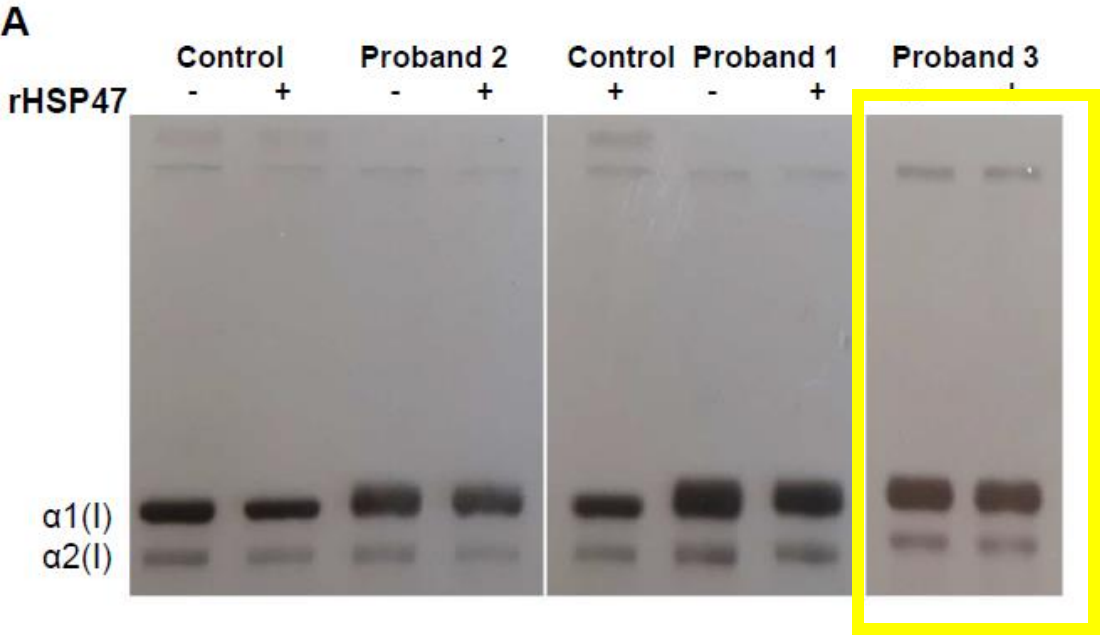

Row image

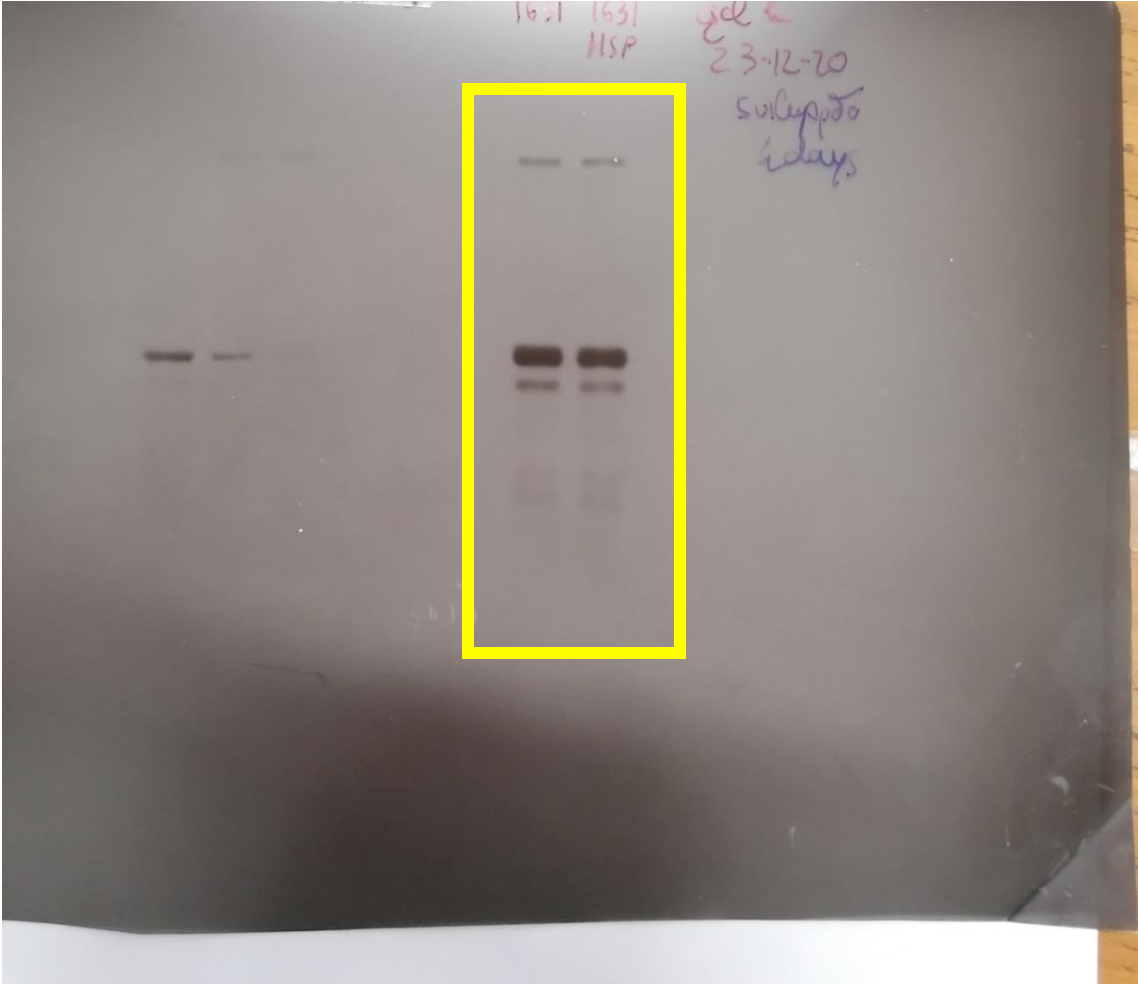

Supplement: Unedited blot and gel images [file jciinsight-10-181570-s116.pdf]
