# Supplementary material for: The administration of exogenous HSP47 as a collagen-specific therapeutic approach
Source: JCI Insight. 2025 Feb 6;10(6):e181570. doi: 10.1172/jci.insight.181570 (PMC11949040; doi:10.1172/jci.insight.181570)
Supplement: Supplemental table 2 [file jciinsight-10-181570-s118.pdf]

Table S2

Figure 2B	IF_Collagen		F_{ARA}= 18.06 P<0.001***				
				Mean (sd)	n	Significance	p-value
		Control	rHSP47+ vs. rHSP47-	730.74(578.03) vs. 114.34(107.98)	n=8, n=8	*	P=0.0460
		Proband 1	rHSP47+ vs. rHSP47-	319.96(191.24) vs. 1000.05(550.94)	n=8, n=8	**	P=0.0063
		Proband 2	rHSP47+ vs. rHSP47-	242.19(148.23) vs. 2104.76(1136.72)	n=8, n=8	***	P<0.001
		Proband 3	rHSP47+ vs. rHSP47-	2104.76(1136.72) vs. 1878.13(777.98)	n=8, n=8	***	P<0.001
		Control vs. Proband 1	rHSP47-	11.13(8.76) vs. 42.63(9.71)	n=8	***	P<0.001
		Control vs. Proband 2	rHSP47-	11.13(8.76) vs. 54.13(8.66)	n=8	***	P<0.001
		Control vs. Proband 3	rHSP47-	11.13(8.76) vs. 54.00(6.97)	n=8	***	P<0.001

Figure 2C	IF_PDI		F_{ARA}= 15.12 P<0.001***				
		Control	rHSP47+ vs. rHSP47-	784.08(511.28) vs. 387.58(490.98)	n=8, n=8	*	P=0.0357
		Proband 1	rHSP47+ vs. rHSP47-	314.57(148.22) vs. 1092.04(304.16)	n=8, n=8	***	P=0.001
		Proband 2	rHSP47+ vs. rHSP47-	1144.81(534.40) vs. 1825.78(512.80)	n=8, n=8	*	P=0.0274
		Proband 3	rHSP47+ vs. rHSP47-	318.00(180.58) vs. 1527.63(520.18)	n=8, n=8	***	P<0.001
		Control vs. Proband 1	rHSP47-	14.38(18.09) vs. 39.88(8.37)	n=8	***	P<0.001
		Control vs. Proband 2	rHSP47-	14.38(18.09) vs 54.88(8.68)	n=8	***	P<0.001
		Control vs. Proband 3	rHSP47-	14.38(18.09) vs. 49.00(11.44)	n=8	***	P<0.001

Figure 2D	IF_PDI_Collagen	F_{ARA}= 40.35 P<0.001***					
		Control	rHSP47+ vs. rHSP47-	111.7(130.25) vs. 19.53(20.99)	n=8, n=8	*	P=0.0272

		Proband 1	rHSP47+ vs.<br>rHSP47-	*267.11(190.68)<br>vs.<br>1006.60(350.61)*	*n=8,<br>n=8*	**	*P=0.0016*
		Proband 2	rHSP47+ vs.<br>rHSP47-	*350.90(289.35)<br>vs.<br>1689.75(822.62)*	*n=8,<br>n=8*	***	*P=0.001*
		Proband 3	rHSP47+ vs.<br>rHSP47-	*143.88(45.03)<br>vs.<br>1937(605.29)*	*n=8,<br>n=8*	***	*P<0.001*
		Control vs.<br>Proband 1	rHSP47-	*6.5(4.42)<br>vs.<br>45.88(5.86)*	*n=8,<br>n=8*	***	*P<0.001*
		Control vs.<br>Proband 2	rHSP47-	*6.5(4.42)<br>vs<br>54.00(6.79)*	*n=8,<br>n=8*	***	*P<0.001*
		Control vs.<br>Proband 3	rHSP47-	*6.5(4.42)<br>vs.<br>56.63(6.19)*	*n=8,<br>n=8*	***	*P<0.001*
**Figure 3B**	**Thioflavin**	***F_{ARA}= 86.82<br>P<0.001****					
		Control	rHSP47+<br>vs.<br>rHSP47-	*1.33 (1.07)<br>vs.<br>3.34 (2.55)*	*n=7,<br>n=7*	*Ns.*	*P=0.1417*
		Proband 1	rHSP47+<br>vs.<br>rHSP47-	*183.15(121.15)<br>vs.<br>922.3526(169.248)*	*n=7,<br>n=7*	**	*P=0.0017*
		Proband 2	rHSP47+<br>vs.<br>rHSP47-	*11.36(11.30)<br>vs.<br>592.03(160.87)*	*n=7,<br>n=7*	**	*P=0.0017*
		Proband 3	rHSP47+<br>vs.<br>rHSP47-	*89.36(103.18)<br>vs.<br>446.90(109.14)*	*n=7,<br>n=7*	**	*P=0.0027*
		Control vs.<br>Proband 1	rHSP47-	*10.71(5.91)<br>vs.<br>52.43(2.88)*	*n=7*	***	*P<0.001*
		Control vs.<br>Proband 2	rHSP47-	*10.71(5.91)<br>vs.<br>45.14(5.08)*	*n=7*	***	*P<0.001*
		Control vs.<br>Proband 3	rHSP47-	*10.71(5.91)<br>vs.<br>39.86(3.98)*	*n=7*	***	*P<0.001*
Figure 3D	**ER thickness**	Proband 1	rHSP47+<br>vs.<br>rHSP47-	135.99(42.87)<br>vs.<br>347.85(114.00)	*n=30,<br>n=30*	***	MW<br>p<0.001
		Proband 2	rHSP47+<br>vs.<br>rHSP47-	155 (67)<br>vs<br>302 (92)	*n=30,<br>n=31*	***	MW<br>p<0.001
		Proband 3	rHSP47+<br>vs.<br>rHSP47-	131 (58)<br>vs<br>270 (68)	*n=30,<br>n=30*	***	MW<br>p<0.001
Figure 4B	**Cell (%)**						
	Live	Control	rHSP47+<br>vs.<br>rHSP47-	79.14(1.41)<br>vs.<br>84.83(2.60)	*n=3,<br>n=3*	*	MW<br>P<0.04
		Proband 1	rHSP47+<br>vs.	66.74(1.21)<br>vs.	*n=3,<br>n=3*	*	MW<br>P<0.04

			rHSP47-	57.56(1.7)8			
		Proband 2	rHSP47+<br>vs.<br>rHSP47-	79.14 (1.41)<br>Vs.<br>84.83 (2.60)	n=3,<br>n=3	Ns.	MW<br>P=0.1
		Proband 3	rHSP47+<br>vs.<br>rHSP47-	86.72 (0.63)<br>Vs.<br>87.30 (0.60)	n=3,<br>n=3	Ns.	MW<br>P=0.4
	Live	Control vs.<br>Proband 1	rHSP47-	84.83(2.60)<br>vs.<br>57.56(1.78)	n=3,<br>n=3	*	P=0.04
		Control vs.<br>Proband 2	rHSP47-	84.83(2.60)<br>Vs.<br>87.30(0.60)	n=3,<br>n=3	Ns.	P=0.2
		Control vs.<br>Proband 3	rHSP47-	84.83(2.60)<br>Vs.<br>86.86(0.45)	n=3,<br>n=3	Ns.	P=0.7
	Apoptotic	Control	rHSP47+<br>vs.<br>rHSP47-	11.35(0.83)<br>vs.<br>10.35(2.00)	n=3,<br>n=3	Ns.	MW<br>P=0.7
		Proband 1	rHSP47+<br>vs.<br>rHSP47-	20.97(1.12)<br>vs<br>31.29(0.99)	n=3,<br>n=3	*	MW<br>P<0.01
		Proband 2	rHSP47+<br>vs.<br>rHSP47-	9.61 (0.33)<br>Vs<br>9.15 (0.28)	n=3,<br>n=3	Ns.	MW<br>P=0.2
		Proband 3	rHSP47+<br>vs.<br>rHSP47-	10.72 (0.81)<br>vs.<br>11.56 (0.41)	n=3,<br>n=3	Ns.	MW<br>P=0.4
	Apoptotic	Control vs.<br>Proband 1	rHSP47-	10.35(2.00)<br>vs.<br>31.29(0.99)	n=3,<br>n=3	**	MW<br>P=0.004
		Control vs.<br>Proband 2	rHSP47-	10.35 (2.00)<br>vs<br>9.15 (0.29)	n=3,<br>n=3	Ns.	MW<br>P=0.71
		Control vs.<br>Proband 3	rHSP47-	10.35 (2.00)<br>vs.<br>11.56 (0.41)	n=3,<br>n=3	Ns.	MW<br>P=0.7
**Figure 4D**	Live	Control	rHSP47+<br>vs.<br>rHSP47-	89.67(2.90)<br>vs.<br>72.75(0.73)	n=3,<br>n=3	*	MW<br>P<0.049
		Proband	rHSP47+<br>vs.<br>rHSP47-	16.80(1.54)<br>vs.<br>8.53(0.45)	n=3,<br>n=3	*	MW<br>P<0.04
	Live	Control vs.<br>Proband	rHSP47-	72.75(0.73)<br>vs.<br>8.53(0.45)	n=3,<br>n=3	**	MW<br>P=0.04
	Early apoptotic	Control	rHSP47+<br>vs.<br>rHSP47-	8.65(0.14)<br>vs.<br>19.78(1.05)	n=3,<br>n=3	*	MW<br>P<0.049
		Proband	rHSP47+<br>vs.<br>rHSP47-	67.18(3.75)<br>vs.<br>25.16(2.87)	n=3,<br>n=3	*	MW<br>P<0.049
	Early apoptotic	Control vs.<br>Proband	rHSP47-	8.65(0.14)<br>vs.<br>19.78(1.05)	n=3,<br>n=3	*	MW<br>P=0.049
	Late apoptotic	Control	rHSP47+<br>vs.<br>rHSP47-	2.72(0.064)<br>vs.<br>5.43(0.317)	n=3,<br>n=3	*	MW<br>P<0.0463
		Proband	rHSP47+<br>vs.<br>rHSP47-	15.83(2.98)<br>vs.<br>66.45(2.19)	n=3,<br>n=3	*	MW<br>P<0.049
	Late apoptotic	Control vs.<br>Proband	rHSP47-	5.43(0.32)<br>vs.<br>66.45(2.18)	n=3,<br>n=3	*	MW<br>P=0.046

Figure 5A	Secreted collagen	Control	rHSP47+<br>vs.<br>rHSP47-	*158.72(13.83)<br>vs.<br>106.37(16.27)*	*n=3,<br>n=3*	*	*MW<br>P=0.049*
		Proband 1	rHSP47+<br>vs.<br>rHSP47-	*56.82(12.89)<br>vs.<br>34.04(1.32)*	*n=3,<br>n=3*	*	*MW<br>P=0.049*
		Proband 2	rHSP47+<br>vs.<br>rHSP47-	*158.24(24.80)<br>vs.<br>84.75(7.63)*	*n=3,<br>n=3*	*	*MW<br>P=0.049*
		Proband 3	rHSP47+<br>vs.<br>rHSP47-	*76.83(7.38)<br>vs.<br>49.70(3.98)*	*n=3,<br>n=3*	*	*MW<br>P=0.049*
		Control vs.<br>Proband 1	rHSP47-	*106.37(16.28)<br>vs.<br>34.04(1.32)*	*n=3,<br>n=3*	***.	*P<0.001*
		Control vs.<br>Proband 2	rHSP47-	*106.37(16.28)<br>vs.<br>84.75(7.63)*	*n=3,<br>n=3*	*	*P=0.049*
		Control vs.<br>Proband 3	rHSP47-	*106.37(16.28)<br>vs.<br>49.70(3.98)*	*n=3,<br>n=3*	***	*P<0.001*
		KW=17.92 p<0.001					
Figure 5F	Matrix collagen	Control	rHSP47+<br>vs.<br>rHSP47-	*129.32(19.85)<br>vs.<br>123.65(22.95)*	*n=3,<br>n=3*	*Ns.*	*MW<br>P=0.82*
		Proband 1	rHSP47+<br>vs.<br>rHSP47-	*213.05(69.75)<br>vs.<br>72.31(10.98)*	*n=3,<br>n=3*	*	*MW<br>P=0.046*
		Proband 2	rHSP47+<br>vs.<br>rHSP47-	*146.75(19.41)<br>vs.<br>37.81(21.27)*	*n=3,<br>n=3*	*	*MW<br>P=0.049*
		Proband 3	rHSP47+<br>vs.<br>rHSP47-	*51.70(14.33)<br>vs.<br>14.71(9.73)*	*n=3,<br>n=3*	*	*MW<br>P=0.049*
		Control vs.<br>Proband 1	rHSP47-	*123.65(22.95)<br>vs.<br>72.31(10.98)*	*n=3,<br>n=3*	**.	*P=0.017*
		Control vs.<br>Proband 2	rHSP47-	*123.65(22.95)<br>vs.<br>37.81(21.27)*	*n=3,<br>n=3*	***	*P<0.001*
		Control vs.<br>Proband 3	rHSP47-	*123.65(22.95)<br>vs.<br>14.71(9.73)*	*n=3,<br>n=3*	***	*P<0.001*
		KW= 11.207, p=0.0107					
Figure 5G	Matrix collagen	Control	rHSP47+ vs.<br>rHSP47-	*112.02 (3.79)<br>Vs<br>100.26 (1.58)*	*n=3,<br>n=3*	*Ns.*	*MW<br>P=0.1*
		α1(I)G478S	rHSP47+ vs.<br>rHSP47-	*94.15 (3.60)<br>Vs<br>72.84 (1.75)*	*n=3,<br>n=3*	*	*MW<br>P=0.04*
		α1(I)G667R	rHSP47+ vs.<br>rHSP47-	*71.96 (6.21)<br>Vs<br>63.83 (0.78)*	*n=3,<br>n=3*	*Ns.*	*MW<br>P=0.1*
		α1(I)G994D	rHSP47+ vs.<br>rHSP47-	*51.68 (3.35)<br>Vs<br>59.14 (1.02)*	*n=3,<br>n=3*	*Ns.*	*MW<br>P=0.1*
		Control vs.<br>α1(I)G478S	rHSP47-	*100.26 (1.58)<br>Vs.<br>72.84 (1.75)*	*n=3,<br>n=3*	**	*MW<br>P=0.01*
		Control vs.<br>α1(I)G667R	rHSP47-	*100.26 (1.58)<br>Vs.<br>63.82 (0.78)*	*n=3,<br>n=3*	**	*MW<br>P<0.001*
		Control vs.<br>α1(I)G994D	rHSP47-	*100.26 (1.58)<br>Vs.*	*n=3,<br>n=3*	**	*MW<br>P=0.01*

			59.14 (1.02)			
Figure 6B	Snout-operculum length	rHSP47+ vs. rHSP47-	*2.52 (0.40) Vs. 2.45 (0.42)*	*n=40, n=48*	*Ns.*	*MW P=0.4*
	Height at eye	rHSP47+ vs. rHSP47-	*1.87 (0.38) Vs. 1.86 (0.34)*	*n=40, n=40*	*Ns.*	*MW P=0.8*
	Snout-operculum length/ height at eye	rHSP47+ vs. rHSP47-	*1.39 (0.16) Vs. 1.35 (0.17)*	*n=40, n=40*	*Ns.*	*MW P=0.4*
**Figure 6D**	Notochord	rHSP47+ vs. rHSP47-	2.17(0.70) vs. 1.85(0.67)	n=41, n=41	*	MW P=0.031
**Figure 6E**	Ceratohyal bone	rHSP47+ vs. rHSP47-	1.90(0.92) vs. 1.51(0.79)	n=41, n=41	*	MW P=0.039
Figure 6F	Ceratobranchial	rHSP47+ vs. rHSP47-	2.93(0.26) vs. 2.79(0.42)	n=41, n=41	*	MW P=0.049
Figure 6G	Mineral content	rHSP47+ vs. rHSP47-	0.03(0.03) Vs. 0.012(0.02)	n=32, n=32	*	MW P=0.0117
